# Cerebro-cerebellar interactions in nonhuman primates examined by optogenetic functional magnetic resonance imaging

**DOI:** 10.1093/texcom/tgac022

**Published:** 2022-05-25

**Authors:** Naokazu Goda, Taku Hasegawa, Daisuke Koketsu, Satomi Chiken, Satomi Kikuta, Hiromi Sano, Kenta Kobayashi, Atsushi Nambu, Norihiro Sadato, Masaki Fukunaga

**Affiliations:** Division of Cerebral Integration, National Institute for Physiological Sciences, 38 Nishigonaka, Myodaiji, Okazaki, Aichi 444-8585, Japan; Department of Physiological Sciences, SOKENDAI (The Graduate University for Advanced Studies), 38 Nishigonaka, Myodaiji, Okazaki, Aichi 444-8585, Japan; Division of System Neurophysiology, National Institute for Physiological Sciences, 38 Nishigonaka, Myodaiji, Okazaki, Aichi 444-8585, Japan; Laboratory for Imagination and Executive Functions, RIKEN Center for Brain Science, 2-1 Hirosawa, Wako, Saitama 351-0198, Japan; Division of System Neurophysiology, National Institute for Physiological Sciences, 38 Nishigonaka, Myodaiji, Okazaki, Aichi 444-8585, Japan; Department of Physiological Sciences, SOKENDAI (The Graduate University for Advanced Studies), 38 Nishigonaka, Myodaiji, Okazaki, Aichi 444-8585, Japan; Division of System Neurophysiology, National Institute for Physiological Sciences, 38 Nishigonaka, Myodaiji, Okazaki, Aichi 444-8585, Japan; Division of System Neurophysiology, National Institute for Physiological Sciences, 38 Nishigonaka, Myodaiji, Okazaki, Aichi 444-8585, Japan; Department of Neurophysiology, National Institute of Neuroscience, National Center of Neurology and Psychiatry, 4-1-1 Ogawa-Higashi, Kodaira, Tokyo 187-8502, Japan; Department of Physiological Sciences, SOKENDAI (The Graduate University for Advanced Studies), 38 Nishigonaka, Myodaiji, Okazaki, Aichi 444-8585, Japan; Division of System Neurophysiology, National Institute for Physiological Sciences, 38 Nishigonaka, Myodaiji, Okazaki, Aichi 444-8585, Japan; Division of Behavioral Neuropharmacology, International Center for Brain Science, Fujita Health University, 1-98 Dengakugakubo, Kutsukake, Toyoake, Aichi 470-1192, Japan; Department of Physiological Sciences, SOKENDAI (The Graduate University for Advanced Studies), 38 Nishigonaka, Myodaiji, Okazaki, Aichi 444-8585, Japan; Section of Viral Vector Development, National Institute for Physiological Sciences, 38 Nishigonaka, Myodaiji, Okazaki, Aichi 444-8585, Japan; Department of Physiological Sciences, SOKENDAI (The Graduate University for Advanced Studies), 38 Nishigonaka, Myodaiji, Okazaki, Aichi 444-8585, Japan; Division of System Neurophysiology, National Institute for Physiological Sciences, 38 Nishigonaka, Myodaiji, Okazaki, Aichi 444-8585, Japan; Section of Viral Vector Development, National Institute for Physiological Sciences, 38 Nishigonaka, Myodaiji, Okazaki, Aichi 444-8585, Japan; Division of Cerebral Integration, National Institute for Physiological Sciences, 38 Nishigonaka, Myodaiji, Okazaki, Aichi 444-8585, Japan; Department of Physiological Sciences, SOKENDAI (The Graduate University for Advanced Studies), 38 Nishigonaka, Myodaiji, Okazaki, Aichi 444-8585, Japan; Division of Cerebral Integration, National Institute for Physiological Sciences, 38 Nishigonaka, Myodaiji, Okazaki, Aichi 444-8585, Japan; Department of Physiological Sciences, SOKENDAI (The Graduate University for Advanced Studies), 38 Nishigonaka, Myodaiji, Okazaki, Aichi 444-8585, Japan

**Keywords:** cerebellum, macaque, motor cortex, opto-fMRI, somatotopy

## Abstract

Functional magnetic resonance imaging (fMRI) is a promising approach for the simultaneous and extensive scanning of whole-brain activities. Optogenetics is free from electrical and magnetic artifacts and is an ideal stimulation method for combined use with fMRI. However, the application of optogenetics in nonhuman primates (NHPs) remains limited. Recently, we developed an efficient optogenetic intracortical microstimulation method of the primary motor cortex (M1), which successfully induced forelimb movements in macaque monkeys. Here, we aimed to investigate how optogenetic M1 stimulation causes neural modulation in the local and remote brain regions in anesthetized monkeys using 7-tesla fMRI. We demonstrated that optogenetic stimulation of the M1 forelimb and hindlimb regions successfully evoked robust direct and remote fMRI activities. Prominent remote activities were detected in the anterior and posterior lobes in the contralateral cerebellum, which receive projections polysynaptically from the M1. We further demonstrated that the cerebro-cerebellar projections from these M1 regions were topographically organized, which is concordant with the somatotopic map in the cerebellar cortex previously reported in macaques and humans. The present study significantly enhances optogenetic fMRI in NHPs, resulting in profound understanding of the brain network, thereby accelerating the translation of findings from animal models to humans.

## Introduction

In systems neuroscience, understanding how neuronal signals in a specific brain region are processed and transferred to the target regions through neural circuits is fundamental. Electrical stimulation and electrical activity recording have been used to investigate such neural circuits. Electrophysiological methods can map neuronal activity with high spatial and temporal resolution but are not adept at mapping large or whole-brain regions. Functional magnetic resonance imaging (fMRI), which measures neuronal activity as blood-oxygen-level-dependent (BOLD) signals, is a promising alternative approach since it simultaneously scans whole-brain regions. However, applying conventional electrical stimulation to brains under fMRI is challenging since electrical stimulation, together with metal stimulating electrodes and wires, induces large electrical and magnetic artifacts in magnetic resonance imaging (MRI). Optogenetics, which can control neuronal activity by light application, is considered to be free from electrical and magnetic artifacts and, therefore, has been used in combination with fMRI (optogenetic fMRI [opto-fMRI]) (Lee et al. 2010; Desai et al. 2011; Lin et al. 2016). Moreover, optogenetics can manipulate a specific cell type with high temporal resolution and is now an indispensable tool for studying brain function in rodents.

The use of nonhuman primate (NHP) models is inevitable for understanding the intricate neuronal mechanisms underlying higher brain functions in humans since they share the following features: homologous brain structures, higher brain functions, precise movement control, perception, and cognition, and similar symptoms in disease states. However, optogenetic application to NHPs has been limited (Inoue et al. 2021; Klink et al. 2021), and urgent development is imperative. Recently, we overcame these obstacles by developing a viral-vector transfection system to strongly express channelrhodopsin-2 (ChR2), precisely targeting layer 5 of the primary motor cortex (M1) based on electrophysiological mapping, and by applying higher intensity laser light to the M1 (Watanabe et al. 2020). Optogenetic intracortical microstimulation (opto-ICMS) evoked sufficient neuronal activity in the M1 of macaque monkeys to induce forelimb movements and muscle activity comparable to those induced by electrical ICMS. This study inspired us to employ our optogenetic stimulation method for opto-fMRI in NHPs for which only a few successful cases have been reported (Gerits et al. 2012; Ohayon et al. 2013; Ortiz-Rios et al. 2021). Herein, we aimed to visualize the brain networks causally influenced by optogenetically induced M1 activation in macaque monkeys using fMRI.

## Materials and methods

### Animals

Two adult female Japanese monkeys (*Macaca fuscata*; monkey C, 5.8 kg body weight; monkey N, 5.0 kg) were used. The experimental protocols were approved by the local Institutional Animal Care and Use Committee, and experiments were conducted according to the guidelines of the National Institutes of Health Guide for the Care and Use of Laboratory Animals. The body weight and food intake were monitored daily during the experimental session. High-resolution MRI was performed before surgery.

### Surgery

Each monkey received a surgery to fix its head painlessly in a stereotaxic frame attached to a monkey chair. After anesthesia with ketamine hydrochloride (5–8 mg/kg body weight, intramuscular [i.m.]) and xylazine hydrochloride (0.5–1 mg/kg, i.m.), propofol (6–9 μg/mL blood concentration) was intravenously injected using a target-controlled infusion pump (TE-371, Terumo) with fentanyl (2–5 μg/kg, i.m.). Following the skin incisions, the skull was widely exposed and the periosteum was completely removed. Small polycarbonate screws were attached to the skull as anchors. The exposed skull and screws were completely covered with transparent acrylic resin. Two polyether ether ketone (PEEK) pipes were mounted in parallel over the frontal and occipital regions for head fixation. All the surgical procedures were performed under aseptic conditions. Arterial oxygen saturation and heart rate were continuously monitored during the surgery. The depth of anesthesia was assessed based on the heart rate and body movements. Additional anesthetic agents were administered when necessary. Antibiotics and analgesics were administered intramuscularly after surgery.

### Mapping of the M1 and injection of a viral vector

After full recovery from surgery, each monkey was seated quietly in a monkey chair with its head painlessly fixed in a stereotaxic frame using PEEK pipes. The skull over the left central sulcus was removed under anesthesia with ketamine hydrochloride (8 mg/kg, i.m.), and the forelimb and hindlimb regions of the M1 were identified by electrophysiological mapping (Nambu et al. 2000; Watanabe et al. 2020). Briefly, a glass-coated tungsten microelectrode (380-120605-00, 0.5 MΩ, Alpha Omega) was inserted perpendicularly to the cortical surface with lidocaine application, and neuronal activity in response to somatosensory stimuli was examined. Then, electrical ICMS (monopolar stimulation, 12 cathodal pulses of 0.2-ms duration at 333 Hz, <50 μA strength) was performed, and evoked movements in the right side of the body were observed.

According to the mapping, an adeno-associated virus (AAV) vector carrying the CAG-hChR2(H134R)/EYFP (Watanabe et al. 2020; 4.0 × 10^12^ viral genome/mL) was injected into the M1 (for details, see Watanabe et al. 2020). The glass micropipette with a Teflon-coated tungsten wire electrode inserted for neuronal recording was connected to a 25-μL Hamilton microsyringe filled with Fluorinert (FC-3283, Sumitomo 3M) using a Teflon tube (JT-10, Eicom), and the AAV solution was loaded from the micropipette. The glass micropipette was inserted into the forelimb or hindlimb region of the M1 perpendicular to the cortical surface through a small incision in the dura mater with local lidocaine application, and the injection sites were selected in putative layer 5 by recording large amplitudes of neuronal spikes. The AAV solution (1 μL at each site) was injected slowly (50 nL/min) using a syringe pump (Nano Jet, Isis), and the micropipette was left in place for an additional 10 min before withdrawing. Injection was performed at two sites in each track: 1 distal forelimb, 1 proximal forelimb, and 2 proximal hindlimb tracks in monkey C (8 μL in total) and 2 distal forelimb, 1 proximal forelimb, and 2 distal hindlimb tracks in monkey N (10 μL in total) (Supplementary Table 1). After the injections, the opened area was completely covered with acrylic resin.

### Implantation of the optical fibers

We used 3 types of optical fibers of 12-mm length, different diameters, and different tip shapes for light stimulation: (i) a 100-μm diameter silica core and flat end (100FT; MFC_100/120-0.22-12 mm_ZF1.25(G)-FLT, Doric), (ii) a 200-μm diameter silica core and flat end (200FT; FG200LEA, Kyocera), and (iii) a 200-μm diameter silica core and cone-shaped end (200CS; MFC_200/240–0.22-12 mm_ZF1.25(G)_C60, Doric). The other end of the optical fiber was equipped with a ferrule and was connected to an optical fiber cable (MFP_100/125/900-0.22_9m_FC-ZF1.25 or MFP_200/220/900-0.22_9m_FC-ZF1.25, Doric). Blue light (473 nm) was delivered through the optical fiber cable by a solid-state blue laser source (100 or 200 mW power output; COME2-473-100LS or COME2-473-200LS, Lucir). The intensity and pattern of the light were controlled using a stimulator (SEN8201, Nihonkohden). Light intensity was measured at the tip of the optical fiber using a power and energy meter (Ophir Optronics) and was expressed as power (mW) or power per unit area (mW/mm^2^).

Three weeks following the AAV injections, each monkey was positioned in the stereotaxic apparatus with its head painlessly restrained under anesthesia with ketamine hydrochloride (8 mg/kg, i.m.), and the acrylic resin over the AAV injection sites was removed. An optical fiber was inserted into the M1 perpendicular to the cortical surface at a horizontal distance of 0.5 mm from the AAV injection track. Repetitive light stimulation (10 pulses of 2-ms duration at 100 Hz, 15 mW intensity, corresponding to 1,911 mW/mm^2^ for 100FT, 478 mW/mm^2^ for 200FT, and 239 mW/mm^2^ for 200CS) was applied every 500 μm in depth, and evoked body movements were observed. The optical fiber was fixed with acrylic resin where light stimulation induced movements (Supplementary Table 1). In the cases where no obvious body movements were induced by light stimulation, the tip of the optical fiber was placed 0.5 mm above the AAV injection sites and fixed. In the control experiments, 2 optical fibers were implanted into the forelimb region of the right M1 of monkey N following electrophysiological mapping, where the AAV vector was not injected (Supplementary Table 1). After optical fiber implantation, 2 acetal resin rectangular chambers for housing the implanted optical fibers were fixed with acrylic resin, and the PEEK pipes for head fixation were removed to reduce the MRI artifacts.

### MRI experiments

In the MRI sessions, each monkey was initially anesthetized with ketamine hydrochloride (5 mg/kg, i.m.) and xylazine hydrochloride (0.6–0.8 mg/kg, i.m.) and was then maintained with continuous intravenous injection of propofol during MRI scans (3–6 μg/mL blood concentration for fMRI, 6–8 μg/mL for structural MRI). The anesthesia condition was adopted to allow stable MRI scans for a long time (Matsui et al. 2011, 2012) and to suppress the limb movements evoked by opto-ICMS for minimizing the secondary BOLD responses caused by the movements (Sultan et al. 2012). The monkeys were placed in the supine position on an acrylic monkey coil holder (Takashima Seisakusho Co. Ltd). In the structural MRI sessions, the head was fixed painlessly with pads and tape inside the array coil. In the fMRI sessions, the head was rigidly fixed to the acrylic monkey coil holder using a custom-made plastic head-post attached to the chamber. The implanted optical fibers were connected to a solid-state blue laser source (COME2-473-100LS or COME2-473-200LS), which was located outside the MRI room, through optical fiber cables (MFP_100/125/900-0.22_9m_FC-ZF1.25 or MFP_200/220/900-0.22_9m_FC-ZF1.25). During all the MRI sessions, vital signs, such as arterial oxygen saturation, heart rate (7500FO, Nonin), respiratory rate (TSD110-MRI, BIOPAC), and body movements (an infrared camera), were continuously monitored using MR-safe systems. The body temperature was maintained using a blanket and hot water bags.

Whole-brain BOLD fMRI activities during opto-ICMS were measured using a block design started with a 15-s initial no-stimulation block followed by alternating 15-s blocks with light stimulation and no stimulation (20 cycles, total 615 s/run). Each fMRI session consisted of 12–21 runs. During the stimulation blocks, 0.5-s light pulse trains (10 pulses of 5-ms duration at 20 Hz, 18–20-mW intensity, corresponding to 2,293–2,548 mW/mm^2^ for 100FT, 573–637 mW/mm^2^ for 200FT, and 287–319 mW/mm^2^ for 200CS) were applied every 1 s (interleaved with 0.5 s interval) unless otherwise stated. These stimulation parameters were adjusted such that apparent thermal fMRI artifacts were not produced during preliminary fMRI sessions for monkey C in which a forelimb region of the M1 was stimulated with several sets of pulse parameters (frequency, duration, and pulse on–off intervals; Supplementary Fig. 1).

The main fMRI experiments targeted 3 stimulation sites in the M1, distal forelimb (digit and wrist), proximal forelimb (elbow and shoulder), and hindlimb (hip or toe) regions for each monkey (Supplementary Table 1). For stimulation of the distal or proximal forelimb region, a single site was stimulated using a single optical fiber, whereas for the hindlimb regions, 2 sites were simultaneously stimulated using 2 optical fibers because the single-site stimulation evoked relatively weak activity in the preliminary fMRI experiment. Within each run, the same site was stimulated, and the stimulation sites varied across the runs/sessions. In total, the data for each stimulation site were collected from 8 to 20 runs across 1–2 sessions: monkey C, distal forelimb (wrist, 100FT) from 16 runs in 2 sessions, proximal forelimb (elbow, 100FT) from 12 runs in 2 sessions, and proximal hindlimb (trunk/hip; 2 200FT) from 8 runs in 1 session; monkey N, distal forelimb (digit/wrist, 200CS) from 20 runs in 2 sessions, proximal forelimb (elbow/shoulder, 100FT) from 20 runs in 2 sessions, and distal hindlimb (hip/toe, 200FT and 100FT) from 8 runs in 1 session. These main fMRI sessions were conducted during 7–20 weeks following AAV injections.

Two additional control fMRI experiments were performed. To examine the effects of light intensity, light with different intensities was applied to the proximal forelimb region in the left M1 of monkey N (10 pulses of 5-ms duration at 20 Hz; 4, 9, and 18 mW intensities corresponding to 510, 1,147, and 2,293 mW/mm^2^, respectively, for 100FT; 12 runs in 2 sessions for each intensity condition). To examine the effect of light on non-AAV vector injection regions, light stimulation was performed in the right M1 of monkey N with the same pulse conditions as those used in the main experiments (10 pulses of 5-ms duration at 20 Hz; 18 mW intensity corresponding to 2,293 mW/mm^2^ for 100FT and 287 mW/mm^2^ for 200CS; 12 runs each) during 24–28 weeks following AAV injections.

### MRI acquisition

MRI was conducted using a 7-tesla whole-body clinical MR scanner (Magnetom 7T, Siemens) with a single-channel volume transmission coil and a multiarray receive coil designed for macaque brain imaging (Takashima Seisakusyo Co. Ltd). The use of the clinical scanner allowed the utilization of advanced imaging techniques developed for human studies. For high-resolution structural images, a 24-channel (monkey N) (Autio et al. 2020) or 4-channel (monkey C) coil was used. A set of the structural images with 0.5-mm isotropic resolution was acquired, including the T2-weighted (T2w) images with a sampling perfection with application optimized contrast using different angle evolutions (SPACE) sequence (repetition time [TR]/echo time [TE], 3,500/291 ms; bandwidth, 651 Hz/Px; flip angle [FA], 120°; averages of 2–3 scans; vendor-provided prototype sequence), T1-weighted (T1w) images with a 3D magnetization-prepared rapid gradient-echo (MPRAGE) sequence (monkey N; TR/TE, 2,500/2.23 ms; inversion time [TI], 1,100 ms; bandwidth, 651 Hz/Px; FA, 5°; averages of four scans), and a 3D magnetization-prepared two rapid gradient-echo (MP2RAGE) sequence (monkey C; TR/TE, 6,000/2.23 ms; TI, 800/2,700 ms; bandwidth, 651 Hz/Px; FA, 5°; averages of 3 scans; vendor-provided prototype sequence).

All the fMRI experiments were performed using a 4-channel receive coil, which has a cylindrical shape suited for placing optical fiber cables for stimulation. BOLD signals were acquired with gradient-echo T2^*^ echo-planar imaging (EPI) sequence using the following parameters: voxel size, 2.0 mm isotropic; axial 28 slices; TR/TE, 750/12.8 ms; field of view (FOV), 128 × 112 mm; phase-encoding direction, posterior-to-anterior (PA); generalized autocalibrating partially parallel acquisitions (GRAPPA) acceleration factor, 2; bandwidth, 2,298 Hz/Px; FA, 30°. The relatively large voxel size was adopted for giving priority to maximizing BOLD sensitivity, and GRAPPA with a short TE was employed to minimize susceptibility-induced signal dropouts and geometric distortions (see Supplementary Fig. 1A for the temporal signal-to-noise ratio of the fMRI data). In addition, a pair of spin-echo EPIs (TR/TE, 4,000/22 ms; FA, 90°; same geometry as the gradient-echo EPI) with opposite phase-encoding directions (PA and anterior-to-posterior [AP]) were acquired for the geometric distortion correction and bias field correction. Further, T2w images with a 2D turbo spin-echo sequence (voxel size, 0.5 × 0.5 × 1.0 mm; axial 56 slices; or 0.5 × 0.5 × 2.0 mm, axial 28 slices; TR/TE, 7,500/85 ms; FOV, 128 × 112 mm; phase-encoding directions, PA and AP; bandwidth, 444 Hz/Px; FA, 60°), T2w image with the 3D SPACE sequence, or T1w image with the 3D MPRAGE sequence were acquired in most fMRI sessions. At the beginning of each session, manual shimming and B1 transmission field map acquisitions were conducted to optimize the shim and transmit power.

### MRI data preprocessing

MRI and fMRI data were analyzed with a customized version of the HCP-NHP pipeline (Autio et al. 2020), FSL v6.0, Freesurfer v5.3.0-HCP, Connectome workbench v1.4.2, AFNI v20.3, and MATLAB.

High-resolution T1w and T2w structural images obtained before surgery were nonlinearly aligned to a representative structural image acquired during fMRI sessions with FSL FLIRT/FNIRT (Smith et al. 2012) to compensate the partial deformation following surgery. These images were then processed with HCP-NHP StructurePipelines (Autio et al. 2020) for alignment to AC-PC space, bias field correction, brain extraction, Freesurfer surface reconstruction, and nonlinear normalization to Yerkes19 (v1.2) template space.

fMRI data were preprocessed with a customized HCP-NHP GenericfMRIVolumePipeline (Autio et al. 2020). The first 8 volumes (6 s) of the fMRI data for each run were removed from the analysis. The data were corrected for gradient nonlinearity distortion and susceptibility-induced distortion using a FSL TOPUP (Smith et al. 2012) method utilizing a pair of spin-echo EPIs with opposite phase-encoding directions. The data were then corrected for head motion and rigidly registered to the high-resolution T1w structural image. For this step, the pipeline was customized to estimate the head motion parameters using images within a brain mask to minimize the potential influence of slow eye drift. In addition, a procedure for the EPI-T1w registration was modified to utilize a T2w structural image acquired within the same fMRI session to improve registration stability. The within-session T2w structural image was corrected for geometrical distortion in the frequency-encoding directions using FSL TOPUP. fMRI data were further processed with the pipeline for spin-echo-based bias field correction and nonlinear normalization to Yerkes19 space with one-step resampling to 1.25-mm isovoxel size (Autio et al. 2020). It should be noted that, despite these efforts on distortion correction and registration, some degree of misalignment between the EPI and T1w structural image persisted in the regions with high distortion, such as those near the chambers. The resultant fMRI data in the NIfTI volume data format were processed with HCP-NHP GenericfMRISurfacePipeline (Autio et al. 2020) to generate CIFTI surface-based data.

### fMRI data analysis

**Fig. 1:**
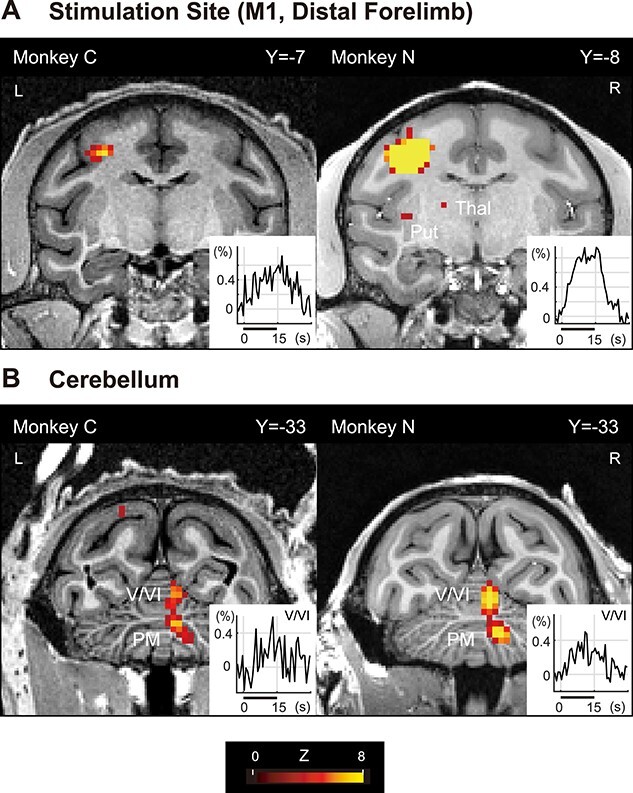
Direct and remote fMRI activations evoked by opto-ICMS of the M1. A and B) *Z*-statistic color maps (stimulation vs. no-stimulation, *P* < 0.05; FWE-corrected for multiple comparisons) were overlaid onto coronal T1w structural images centered at the local maxima in the M1 (A) and cerebellum (B). The distal forelimb region in the left M1 was stimulated focally in monkey C (left) or widely in monkey N (right). Y represents the coordinates in the Yerkes19 macaque template space with the anterior commissure as the origin. Insets show time courses of the mean signal (percent signal changes) at the local maxima in the M1 (A) or lobules V–IV of the cerebellum (B) during (horizontal bar, 15 s) and after the stimulation block. The local maxima were defined using data from the odd runs, and the time courses were evaluated from the even runs. V, VI, cerebellar lobules V and VI; PM; Put, putamen; Thal, thalamus; L, left; R, right.

Whole-brain statistical analyses were performed using FSL FEAT (Smith et al. 2012) with HCP-NHP TaskfMRIPipeline (Autio et al. 2020). The preprocessed fMRI data were temporally high-pass filtered (cutoff, 60 s), spatially smoothed (full width at half maximum, 2 mm), and then tested with a first-level general linear model (GLM) for each run. The regressor was a boxcar function representing stimulation/no-stimulation alternating blocks convolved with the gamma hemodynamic response function (lag, 4.5 s; SD, 2 s) optimized for macaques (Grohn et al. 2020). Statistical significance of the activity for each stimulation site was subsequently tested with a second-level, within-subject, fixed-effects GLM analysis on the first-level results from all available runs (1-tailed one-sample *t*-test). The resultant *t*-statistics were converted to *z*-statistics and were corrected for multiple comparisons (*P* < 0.05, family-wise error [FWE] rate correction based on random field theory). The BOLD response magnitude (beta weights in the first-level analysis) and average time course aligned at the onset of the stimulation block (Fig. 1 insets and Supplementary Fig. 1C) were extracted from the voxel at the local maxima of the *z*-statistic map defined using data from independent runs (Fig. 1 and Supplementary Fig. 1). The time course was represented as percent signal change for which a baseline was computed from the signals of the four time-points before the onset of stimulation blocks. These GLM analyses were performed on NIfTI volume and CIFTI surface data separately: Cerebral cortex panels in Figure 3A and B represent the surface data analysis results, and the others show volume data analysis results.

### Atlas registration, cerebellum segmentation, and region of interest definitions

The high-resolution structural image in the Yerkes19 space was nonlinearly aligned to the NIMH Macaque Template (NMT) v2.0 template with AFNI @animal_warper (Jung et al. 2021) for registration of the Subcortical Atlas of the Rhesus Macaque (SARM) level 6 atlas (Hartig et al. 2021). The cerebellum was extracted by applying the NMT cerebellum mask warped to each subject’s brain. The segmented cerebellum and fMRI activations were volume-rendered using MRIcron (https://people.cas.sc.edu/rorden/mricron/) in the superior and inferior views (maximum-intensity projection; search depth, 8 voxels [4 mm]; background/overlay transparency, 50%). To avoid confusion concerning the superiorly and inferiorly located activations in these views, activations only in the superior part of the cerebellar cortex, consisting of lobules III–VII, the simple lobule, and lateral hemisphere (Cb3v-7v, Cb3I-Cb7I, CbSim, and Crus1-2 in the SARM atlas), were displayed in the superior view, and activations in the inferior part, consisting of lobules I–II, VII–X, paramedian lobule (PM), copula of the pyramids, and the lateral hemisphere (Cb1v-2v, Cb7v-10v, CbPM, CbCop, and Crus1-2), were displayed in the inferior view. Each SARM atlas area warped to monkey N’s brain was also volume-rendered on the segmented cerebellum (Figs 3C and 4D; the same settings described above). We defined the anterior lobe as consisting of lobules I–VI based on the study by Boillat et al. (2020).

For anatomical region of interest (ROI) analysis, 19 cerebellar cortex ROIs (Cb1v-10v, Cb3I-Cb6I, CbSim, CbPM, CbCop, CbCrus1, and CbCrus2) were defined based on the SARM level6 atlas. Voxel-wise magnitudes of the BOLD responses (beta weights in the first-level GLM results) were averaged within each ROI for each run, then averaged across multiple runs, and statistically tested with a 1-tailed one-sample *t*-test for each subject using MATLAB (*P* < 0.05, corrected for multiple comparisons using Bonferroni correction).

Additionally, M1 and cerebellum functional ROIs in monkey N were defined as significant clusters (*P* < 0.05, FWE-corrected for multiple comparisons using random field theory) around the stimulation sites and in the cerebellum from the main results targeting the proximal forelimb. Data from the control fMRI experiments were analyzed using these functional ROIs as described above for anatomical ROIs.

### Ex vivo MRI and histology

One year following the first AAV injection, monkey N was deeply anesthetized with sodium thiopental (50 mg/kg, intravenous) and was perfused transcardially with 0.1 M phosphate buffer (PB) followed by 10% formalin in 0.1 M PB, and then 0.1 M PB. The brain was removed immediately and stored in saline at 4 °C.

The fixed brain for monkey N was scanned ex vivo with MRI using the 24-channel multiarray receive coil. The brain was placed in a container filled with saline and positioned using Teflon sheets. Air bubbles in the sample were mostly removed by placing the sample under a negative vacuum pressure overnight. Three T2w images with 0.25-mm isotropic resolution (SPACE; FOV, 80 × 80 mm; matrix size, 320 × 320; axial 256 slices; TR/TE, 4,500/448 ms; bandwidth, 372 Hz/Px; FA, 120°; number of averages, 4) were acquired consecutively. The total scanning time was approximately 5 h.

The ex vivo structural images were processed using ANTs v2.3.1 (Avants et al. 2010). Three T2w images were bias-corrected using ANTs N4BiasFieldCorrection and were nonlinearly aligned and averaged using antsMultivariateTemplateConstruction2.sh. The average T2w image was then nonlinearly registered with the in vivo T1w image in Yerkes19 space using antsRegistrationSyN.sh. Then the transformation for the registration between the ex vivo and in vivo images was applied to the fMRI results to map them onto the ex vivo image space.

After performing the ex vivo MRI, the brain was saturated with 0.1 M PB containing 30% sucrose and was then cut into frontal 60-μm thick sections on a freezing microtome. Two of every 5 free-floating sections were incubated overnight with rabbit anti-GFP primary antibody (1:1,000; Thermo Fisher Scientific) in 0.01 M phosphate buffered saline at 4 °C. Subsequently, the sections were visualized with secondary antibody conjugated with Alexa Fluor 488 (1:1,000; Thermo Fisher Scientific) for 2 h at room temperature. One of the 2 groups were additionally stained with red fluorescent Nissl (NeuroTrace 530/615; Thermo Fisher). They were subsequently mounted onto gelatin-coated glass slides and cover-slipped with antifade mounting medium (Vectashield, Vector Laboratories). Images of the brain sections were captured using a digital fluorescence microscope (BZ-X710, Keyence).

## Results

### Opto-ICMS of the M1

An optical fiber was implanted in the vicinity of each AAV injection site in the M1 (Supplementary Table 1; distal and proximal forelimb and proximal hindlimb regions in monkey C; distal and proximal forelimb and distal hindlimb regions in monkey N). Postmortem histological examination demonstrated that ChR2 tended to be mainly labeled in the deep layers, such as in and around the layer 5 of the M1 (Supplementary Fig. 2A and B), although other layers were also labeled (Supplementary Fig. 2C). We confirmed that opto-ICMS of the forelimb M1 (473 nm, 10 pulses of 2-ms duration at 100 Hz, 15-mW intensity) successfully induced forelimb movements in both the monkeys under sedation with ketamine hydrochloride as observed in our previous study (Supplemental Movie 1 in the study by Watanabe et al. 2020).

### Robust direct and remote fMRI activation evoked by opto-ICMS of the M1

We then aimed to identify the brain networks activated through opto-ICMS of the M1 with 7-tesla fMRI scans under anesthesia. During the stimulation blocks in the fMRI scans, a light pulse train (473 nm, 10 pulses of 5-ms duration at 20 Hz, 18–20 mW intensity) was applied every 1 s (interleaved with 0.5 s interval). These parameters of light stimulation were adjusted based on the results of the preliminary fMRI sessions conducted before the main experiments (Supplementary Fig. 1).

**Fig. 2:**
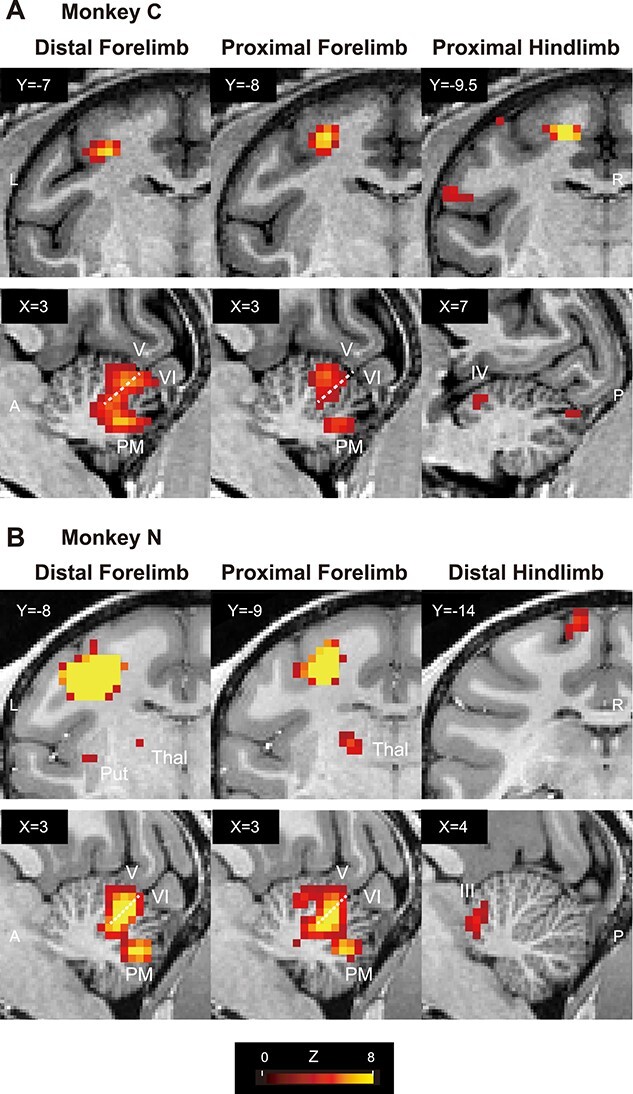
Somatotopic cerebellar activations evoked by opto-ICMS of the M1. A and B) *Z*-statistic color maps (stimulation vs. no-stimulation, *P* < 0.05 FWE-corrected for multiple comparisons) were overlaid onto coronal T1w images of the left M1 (upper row) and sagittal images of the right cerebellum (lower row) for monkey C (A) and monkey N (B). Left, middle, and right panels represent maps with the stimulations of the M1 distal forelimb, proximal forelimb, and hindlimb (A, proximal hindlimb for monkey C; B, distal hindlimb for monkey N) regions, respectively. Left panels (distal forelimb) in (A) and (B) are replots of Figure 1. Dotted lines in the cerebellum denote primary fissure. *X* and *Y* represent the coordinates in the Yerkes19 macaque template space. III–VI, cerebellar lobule III–VI; A, anterior; P, posterior.

We found that the opto-ICMS of the M1 evoked robust activation in the stimulation site and remote brain regions (Fig. 1; see also Supplementary Fig. 3). Light stimulation was applied to the distal forelimb region in the left M1: to the wrist region focally with the 100FT (monkey C, Fig. 1A, left) and to the digit region widely with the 200CS (monkey N, Fig. 1A, right). In both the cases, the left M1 was activated, peaking at the anterior bank of the central sulcus (Fig. 1A; *P* < 0.05, corrected for multiple comparisons). The locations of the activation peaks correspond well to the bottom ends of the optical fiber tracks in the high-resolution ex vivo and in vivo MRI (Supplementary Fig. 4). The activation was more prominent and extended in monkey N, probably in part owing to the wide-area stimulation by a cone-shaped end fiber. In addition to these direct activations at the stimulation site, we found 2 distinct remote activations in the right cerebellar cortex, contralateral to the stimulation site (Fig. 1B). One was in the anterior lobe, peaking at the primary fissure (the vermis of lobules V and VI), and the other was in the posterior lobe, peaking at the PM in the intermediate zone of the cerebellar hemisphere. Activations were also observed in the intermediate zone of lobules IV–V of right cerebellar cortex (Supplementary Fig. 3A and B), left thalamus (Fig. 1A, right; Fig. 2B, upper; Supplementary Fig. 3A and B), and left putamen (monkey N; Fig. 1A, right; Supplementary Fig. 3B).

### Somatotopic organization in the cerebellum revealed by opto-ICMS of the M1

We compared the distribution of the activities derived from the opto-ICMS targeting 4 M1 subregions that represent the distal and proximal forelimbs and the proximal and distal hindlimbs. We first confirmed the activation at these stimulation sites topographically arranged along the anterior bank of the central sulcus in the M1, as expected (Fig. 2A and B, upper panels; Supplementary Fig. 4). In the anterior lobe of the cerebellar cortex of monkey C, the distal forelimb M1 stimulation activated the region around the primary fissure (lobules V and VI; Fig. 2A, distal forelimb, lower panel), whereas the proximal forelimb M1 stimulation activated the anterior region peaking at lobule V (Fig. 2A, proximal forelimb, lower panel), although they overlapped each other. Similar tendencies were observed in monkey N (Fig. 2B, distal forelimb, proximal forelimb, lower panels). Hindlimb M1 stimulation activated more anterior regions around lobules III–IV (Fig. 2A and B, proximal hindlimb, distal hindlimb, lower panels), and the activated region was more anteromedial in monkey N (distal hindlimb M1 stimulation) than in monkey C (proximal hindlimb). In the posterior lobe of the cerebellar cortex, distal, and proximal forelimb M1 stimulation activated the PM, which overlapped each other in both monkeys (Fig. 2A and B, distal forelimb, proximal forelimb, and lower panels), while the hindlimb M1 stimulation induced the activation of the more posterior region in monkey C (Fig. 2A, proximal hindlimb, lower panel) and no activation in monkey N (Fig. 2B, distal hindlimb, lower panel).

**Fig. 3:**
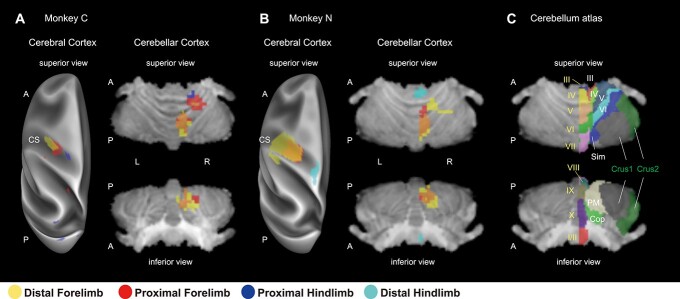
Summary of somatotopic organization in the cerebellum revealed with opto-fMRI. A and B) cerebral cortex: Direct activations at the 4 stimulation sites of the M1 (distal forelimb, proximal forelimb, distal hindlimb, and proximal hindlimb) were surface-rendered on the inflated cerebral cortical surface of the left hemisphere (A, monkey C; B, monkey N) with more conservative statistical criteria (*P* < 10^−4^ uncorrected). Cerebellar cortex: Significant cerebellar activations with the stimulation of the four sites shown in Fig. 2 were volume-rendered in the superior and inferior views (A, monkey C; B, monkey N). In (A) and (B), yellow, red, blue (monkey C only), and cyan (monkey N only) colors denote distal forelimb, proximal forelimb, proximal hindlimb, and distal hindlimb representations, respectively. Orange denotes overlaps between distal and proximal forelimb representations. C) The SARM level6 atlas projected onto the cerebellum for monkey N is shown as a reference of the anatomical subdivisions in the cerebellar cortex. Letters colored with yellow, white, and green denote the regions in the vermis, intermediate zones, and lateral hemispheres, respectively. I–X, cerebellar lobule I–X; Sim, simplex lobule; Cop, copula pyramids; CS, central sulcus.

To obtain an encompassing view of the topography of the optogenetically evoked activations shown in Fig. 2, we superimposed them on the cerebral cortical surface and segmented cerebellum (Fig. 3A and B). In the anterior lobe (Fig. 3A and B, cerebellar cortex, superior view), the overall somatotopic representation was arranged in the anteroposterior direction in the vermis and intermediate zone. The distal forelimb region (yellow) was located most posteriorly; the proximal forelimb region (red), slightly anteriorly, with overlaps to the distal forelimb region (orange); the proximal hindlimb region (blue), more anteriorly; and the distal hindlimb region (cyan), anteriorly and medially. There were some individual variations in the intermediate zone: The proximal forelimb region, as a separate cluster from that in the vermis, was more extended than the distal forelimb region in monkey C, while the distal forelimb region extended laterally in monkey N. These distribution differences reflected, in part, the differences in the location and area of the stimulation site for the distal forelimb: The wrist region was focally stimulated in monkey C, and the digit region was widely stimulated in monkey N (see also the activated regions in the M1, Fig. 3A and B, cerebral cortex). In the posterior lobe of the cerebellum (Fig. 3A and B, cerebellar cortex, inferior view), distal and proximal forelimb regions overlapped in the PM, and no or little hindlimb activation was observed, suggesting no clear somatotopic topography compared with the anterior lobe.

### ROI analysis

**Fig. 4:**
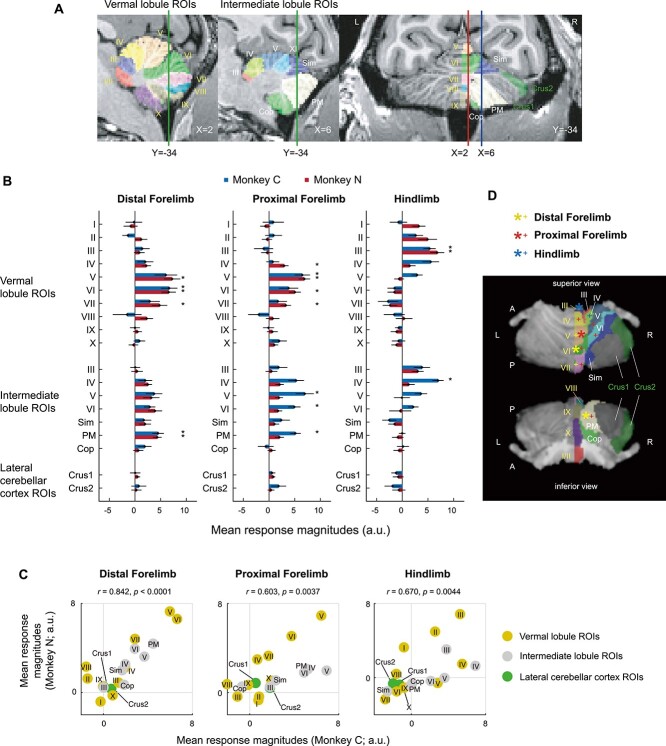
ROI analysis. A) Atlas-based ROIs were shown on the sagittal (left and middle) and coronal (right) T1w images for monkey N. The ROIs were grouped into vermal lobule (left, denoted by yellow letters), intermediate lobule (middle, white letters), and lateral cerebellar cortex (right, green letters) ROIs. Green vertical lines in the left and middle sagittal images denote the coronal plane of the right panel (*Y* = −34), and red and blue vertical lines in the right coronal image denote the sagittal planes of the left (*X* = 2) and middle (*X* = 6) panels, respectively. B) Mean response magnitudes (beta weights) across runs in the 19 ROIs were plotted for the distal forelimb (left), proximal forelimb (middle), and hindlimb (right; proximal, blue; distal, red) region stimulations for each subject (blue, monkey C; red, monkey N). Error bars indicate the standard error of the mean. ^*^  *P* < 0.05, 1-tailed one-sample *t*-test, corrected for multiple comparisons (Bonferroni correction for each stimulation site and subject). C) Mean response magnitudes in 19 ROIs of monkeys C (abscissa) and N (ordinate) were plotted for stimulation of the distal forelimb (left), proximal forelimb (middle), and proximal or distal hindlimb (right) M1. Yellow, gray, and green symbols represent vermal lobule, intermediate lobule, and lateral cerebellar cortex ROIs, respectively. Correlation coefficients (*r*) between the subjects and the statistical significance (*p*; permutation test) are shown in the insets. D) Results of the statistical tests shown in (B) are summarized in the superior and inferior views. Large ^*^ indicates statistically significant activities in both the subjects, and small + indicates significant activities in either single subject. Yellow-, red-, and blue-colored marks represent distal forelimb, proximal forelimb, and hindlimb stimulations, respectively.

We further performed an ROI analysis to quantitatively assess the somatotopic organization in the cerebellar activities described above. We evaluated the BOLD response magnitudes in 19 ROIs on the cerebellar cortex defined based on the SARM level 6 atlas (Hartig et al. 2021) (Figs.3C and 4A). These results provide additional evidence for the anteroposterior gradient of the somatotopic organization in the anterior lobe (Fig. 4B). As seen in the voxel-wise analyses above, consistent with the 2 subjects, the distal and proximal forelimb M1 stimulation evoked significant activities in the vermis of lobules VI and V, respectively (*P* < 0.05, corrected for multiple comparisons). For the hindlimb, stimulation of the proximal (monkey C) and distal (monkey N) sites commonly evoked significant activities in the vermis of lobule III. In the posterior lobe, significant activities were observed in the PM with distal forelimb M1 stimulation in the two subjects. The ROI response profiles were significantly correlated between 2 monkeys for each of the 3 stimulation site conditions regardless of the difference in the exact locations of the stimulation sites (Fig. 4C). This supports the commonalities of the overall topography between the subjects. Figure 4D shows a summary of the results.

### A causal relationship between opto-ICMS and direct/remote fMRI activity

To ensure that the observed activation in the cerebellum was causally related to optogenetically evoked M1 activity, we conducted 2 control fMRI experiments for 1 monkey (monkey N, Supplementary Fig. 5). First, we tested whether remote cerebellar activity is dependent on the intensity of opto-ICMS. We stimulated the proximal forelimb region in the left M1 with 3 light intensities (4, 9, and 18 mW) and compared the magnitudes of the activities in the 2 functional ROIs, the stimulation site and the cerebellum (lobules V–VI and PM), defined from the main results shown in Fig. 2B. We found that the activity magnitudes tended to monotonically increase as the light intensity increased in both the stimulation site and the cerebellum (Supplementary Fig. 5A and B), i.e. manipulating the M1 activity caused corresponding changes in the cerebellar activity. This pattern of results supports a causal relationship between the opto-ICMS of the M1 and cerebellar activity.

Second, to assess the possibility that the thermal artifacts (heating-induced signal change) (Christie et al. 2013; Schmid et al. 2017; Albers et al. 2019) would contaminate the observed activations, the M1 without viral-vector injection was optically stimulated (Supplementary Table 1). In the fMRI experiment, the right M1 of monkey N was optically stimulated by light pulse trains with the same pattern and intensity (18 mW) as those used in the main experiments. Optical stimulation of the proximal forelimb M1 with the 200CS induced no signal changes at the stimulation site (Supplementary Fig. 5C, upper panel, left). Stimulation of the distal forelimb M1 with the 100FT induced focal signal changes at the stimulation site; however, they were much weaker and smaller than those with opto-ICMS of the left M1 (compare Supplementary Fig. 5, A, upper panel, right, and C, upper panel, right). The observed activation might be caused by ChR2 expressed axon terminals of commissural fibers from the left M1. Based on these results, we believe that the effect of heating at the stimulation sites would have only marginal effects on our main results. Furthermore, there was no evident signal change in the cerebellum with light stimulation alone in all the cases (Supplementary Fig. 5C; lower panel). Thus, we reasonably conclude that remote activation observed in the cerebellum was indeed evoked by opto-ICMS of the M1.

## Discussion

In this study, we stimulated the distal and proximal forelimb and hindlimb regions of the M1 in macaque monkeys using optogenetic methods with efficient viral-vector and high-intensity stimulation and observed the BOLD responses in the cerebellum using 7-tesla MRI. We found 2 primary activations in the cerebellar cortex, contralateral to the stimulation site. One was in the anterior lobe, peaking at the vermis and intermediate zone of the cerebellar hemisphere of lobules III–VI, and the other was in the posterior lobe, peaking at the PM. In the anterior lobe of the cerebellum, projections from the distal hindlimb, proximal hindlimb, proximal forelimb, and distal forelimb regions of the M1 were somatotopically arranged in the anteroposterior direction. There was no clear somatotopic topography in the posterior lobe of the cerebellum. This study greatly advances opto-fMRI in NHPs (Gerits et al. 2012; Ohayon et al. 2013; Ortiz-Rios et al. 2021), a challenging but promising method for investigating effective connectivity in vivo.

### Cerebellar activity evoked by opto-ICMS

We identified the BOLD responses in the cerebellar cortex induced by opto-ICMS of the M1. Since the cerebellum does not directly connect with the M1, cerebellar activity reflects signal inputs polysynaptically mediated from the M1 through the precerebellar and inferior olivary nuclei in the brain stem and the mossy and climbing fibers (Kelly and Strick 2003; Coffman et al. 2011). Electrical ICMS of the cerebral cortex in macaque monkeys can evoke cerebellar activity through polysynaptic cerebro-cerebellar projections (Adrian 1943; Snider and Eldred 1952; Sasaki et al. 1977). Sasaki and his colleagues (1977) have demonstrated that electrical stimulation of the macaque M1 induces large long-lasting local field potentials in the cerebellar cortex. Electrophysiological recording identified an early (latency, 4–5 ms) negative mossy fiber response and a late (15–18 ms) negative climbing fiber response in the cerebellar vermis and hemisphere on the side contralateral to the stimulation. Mossy and climbing fiber inputs induce local field potentials and strongly activate neurons, including Purkinje cells. Thus, the BOLD responses observed in this study reflect the activity of these nerve terminals and somata. In addition, electrical ICMS of the primary somatosensory cortex in anesthetized macaques evoked remote BOLD responses in the contralateral cerebellum (Matsui et al. 2011, 2012), and electrical stimulation of the cerebellar nucleus evoked BOLD reponses in the contralateral cerebral cortex (Sultan et al. 2012).

By contrast, the activation of remote brain regions through polysynaptic routes by opto-ICMS in macaques has been unsuccessful. For example, previous studies showed that optogenetic stimulation of the primary visual cortex (V1) evoked activities only in V1 and the areas directly connected to V1 (Nakamichi et al. 2019; Ortiz-Rios et al. 2021). In the present study, we demonstrated that opto-ICMS could involve cerebro-cerebellar interactions, probably owing to the efficient viral vectors and optimized stimulation. This finding provides new evidence that opto-ICMS is as efficient as electrical ICMS to activate polysynaptically connected brain regions.

### Somatotopic organization of the cerebellum

There is somatotopic organization in the cerebellum, with an upside-down map in the anterior lobe and a second representation in the posterior lobe (Manni and Petrosini 2004). Anatomical studies in monkeys have shown that the cells labeled from anterograde or retrograde transsynaptic tracer injections in the M1 forelimb region were richly found around the cerebellar lobules IV–VI and PM (Kelly and Strick 2003; Lu et al. 2007; Voogd 2014). Lu and his colleagues (2007) injected rabies virus into various regions of the M1 of macaque monkeys. The rabies virus was retrogradely and transsynaptically transported to the Purkinje cell through the thalamus and cerebellar nuclei. They found somatotopy along the anteroposterior and lateromedial axes of the cerebellar cortex. Purkinje cell labeling from the forelimb representation, including the proximal and distal regions, was observed primarily in lobules IV–VI of the anterior lobe. Proximal forelimb labeling was both anterior and lateral to that of the distal forelimb within lobules IV–VI. By contrast, hindlimb labeling was seen both anterior and lateral to that of the proximal forelimb within lobules III–VI. A similar topographic map along the anteroposterior axis in the anterior lobe has also been supported by the electrophysiological recordings with electrical ICMS of the M1 (Adrian 1943; Snider and Eldred 1952; Sasaki et al. 1977). Stimulation of the forelimb and face areas of the M1 evoked responses mainly in the posterior part of the anterior lobe and the PM, whereas stimulation of the hindlimb area provoked responses predominantly in the anterior part of the anterior lobe, suggesting somatotopic cerebro-cerebellar projections. The map is also similar to that measured with somatosensory stimulations (Van Kan et al. 1993; Ishikawa, Tomatsu, Tsunoda, Hoffman, et al. 2014a; Ishikawa, Tomatsu, Tsunoda, Lee, et al. 2014b) and is consistent with single-unit recordings during motor tasks (Mano et al. 1980, 1986; Marple-Horvat and Stein 1987; Ishikawa, Tomatsu, Tsunoda, Hoffman, et al. 2014a; Ishikawa, Tomatsu, Tsunoda, Lee, et al. 2014b). Therefore, our large-scale somatotopy map obtained with opto-fMRI was consistent with the findings reported previously, adding new evidence to consolidate the knowledge of cerebellar architecture accumulated over a century (Manni and Petrosini 2004). Moreover, our map is also remarkably similar or consistent to that reported in human fMRI studies that examined activations during motor tasks (Nitschke et al. 1996; Grodd et al. 2001; Takanashi et al. 2003; Thickbroom et al. 2003; Stoodley et al. 2010; Van der Zwaag et al. 2013; King et al. 2019; Boillat et al. 2020) and resting-state connectivity (Buckner et al. 2011; Xue et al. 2021). These similarities support a common somatotopic organization around these cerebellar regions between NHPs and humans. Note that those task-related fMRI activations and resting-state fMRI connectivity do not provide information about the direction of signal flow. This may even be true for fMRI with electrical ICMS, which induces both orthodromic and antidromic activation (Klink et al. 2021). Our opto-fMRI result is unique in identifying the efferent signals from the M1, revealing for the first time the meso-scale topography of the M1 projections in the macaque cerebellum in vivo.

The aforementioned studies in monkeys and humans have reported another anteroposterior gradient of somatotopy in the posterior lobe around the PM (Manni and Petrosini 2004). However, the somatotopic gradient in this region is difficult to obtain compared to that in the anterior lobe: Distal forelimb, proximal forelimb, and hindlimb representations in the posterior lobe intermingled in monkeys (Lu et al. 2007), and those in humans tend to be crude or undetected (Grodd et al. 2001; Mottolese et al. 2013; Van der Zwaag et al. 2013; Boillat et al. 2020). Consistent with these organizations, we did not identify a distinct somatotopic gradient around the PM, although the activation was repeatedly observed, especially with forelimb region stimulation. This result suggests a more indistinct or fractured somatotopic organization in the posterior lobe. One limitation of this study is that we did not test face representation, which would be expected to lay posteriorly to the forelimb region. Further investigation with wider body coverage (Boillat et al. 2020) might clarify the topographic organization of this region in the future.

One intriguing aspect of our results is that the cerebellar regions activated by opto-ICMS of the M1 were circumscribed in the medial part of the cerebellar cortex, vermis, and intermediate lobules. The cerebellar vermis has been traditionally recognized to receive major inputs from the spinal cord (Adrian 1943); however, a recent anatomical study has shown that it also receives dense inputs from the M1 forelimb regions via the pons (Coffman et al. 2011; see also Sasaki et al. 1977). The signal to the vermis during opto-ICMS is routed through the cortico-ponto-cerebellar pathway, although the activity of the pons could not be confirmed using fMRI results, probably owing to its small size. We did not observe activations in the lateral part, where anatomical connections with the M1 are known (Kelly and Strick 2003; Lu et al. 2007). The lack of activity in the lateral cerebellum could be ascribed to the low signal-to-noise ratio in these regions (Supplementary Fig. 1A) and small mossy and climbing fiber responses in these regions evoked by the forelimb and hindlimb M1 stimulation (Sasaki et al. 1977). Besides this, the stimulation site or parameters employed could be unsuitable to activate those regions. Our stimulation behaviorally induced a simple movement but not a complex one. Human fMRI findings indicate that the lateral part of the cerebellum (including a part of lobules VI and I) is involved in complex motor actions, such as sequential finger or toe movements (Schlerf et al. 2010). We thus presume that the stimulations inducing more complex motor behaviors (Graziano et al. 2002; Hira et al. 2015) could engage the lateral cerebellar cortex.

### Cortico-cortical and cortico-subcortical transmission of optogenetically evoked signals

Our results unexpectedly demonstrated that opto-ICMS of the M1 did not efficiently activate the motor-related cortices that are densely connected to the M1. We suppose this to be attributed to the suboptimal conditions for effectively inducing cerebral cortico-cortical interactions. First, electrical ICMS under anesthesia often suppresses the cerebral cortico-cortical signal transmission (Logothetis et al. 2010; Matsui et al. 2012). We suggest that similar anesthesia-induced cerebral cortico-cortical suppression occurs during opto-ICMS. Anesthesia not only suppresses but also enhances stimulus-driven and resting neuronal activities in some regions owing to the release from inhibition (Uhrig et al. 2016). Thus, the effect of anesthesia is not uniform across the brain resulting in imbalanced cortico-cortical, cortico-subcortical, and intra-/inter-hemispheric functional connectivity patterns (Becq et al. 2020). Next, stimulation frequency may also have an impact on the observed remote activations (Chan et al. 2020; Murris et al. 2020; Ortiz-Rios et al. 2021). Electrical ICMS in macaques (Murris et al. 2020), as well as opto-ICMS in rat (Chan et al. 2020), have reported that remote fMRI activations were frequency-dependent and could be even negative under some conditions. Lastly, the pattern of the remote activations may be dependent on the stimulated cortical layer (Chan et al. 2020; Ortiz-Rios et al. 2021). Particularly, a recent study has reported that layer-specific optogenetic stimulation of different layers in rat M1 evoked distinct patterns of remote fMRI responses (Chan et al. 2020). In the present study, opto-ICMS targeted to the layer 5 (Supplementary Fig. 2), and the layer 5 neurons, which are the origins of cortico-ponto-cerebellar (Coffman et al. 2011) and cortico-striatal projections, may have been most effectively excited, whereas the layer 2 and 3 neurons projecting to other cerebral cortices may have been less excited. Meanwhile, the lack of activation in the cerebral motor cortices, regardless of the underlying mechanism, naturally indicates that the remote cerebellar activity was driven by the M1 and not through the other cerebral cortices.

The activation was also not reliably detected in subcortical structures, such as the basal ganglia (Bostan et al. 2013). Exceptions were the thalamus (Figs 1 and 2; Supplementary Fig. 3) and putamen (Figs 1 and 2; Supplementary Fig. 3), which may be activated through the M1-thalamic or cerebellar cortico-cerebellar nuclei-thalamic projection and through the M1-putamen projection, respectively. Lack of reliable activation in the subcortical structures is not explained by cerebral cortico-cortical suppression or cortical layer specificity. Other factors should be considered, such as complex response patterns composed of excitation and inhibition in the basal ganglia evoked by M1 stimulation (Nambu et al. 2000). It is noteworthy that earlier opto-ICMS of rat M1 produced remote fMRI responses, including those in the basal ganglia, more reliably with an optimized fMRI sequence (passband-balanced steady-state free precession) than with a conventional gradient-echo BOLD sequence (Lee et al. 2010). Thus, whether measurements with higher spatial resolution and sensitivity under further optimized fMRI parameters can detect optogenetically evoked activity in motor-related cerebral cortices and subcortical structures is a pertinent question. More works need to be done to optimize high-field opto-fMRI in NHPs.

In summary, our study convincingly demonstrated that opto-ICMS of macaque M1 effectively evoked somatotopic cerebellar activations via the polysynaptic cerebro-cerebellar pathway. With this technique, we revealed for the first time, meso-scale somatotopic map of the M1 projections in the cerebellum, which could not be obtained with other in vivo methods. The somatotopy was consistent with those reported with anatomical tracing and electrophysiology in monkeys and neuroimaging in humans, bridging those knowledges across human and NHPs and adding evidences on common organization of cerebro-cerebellar interactions across species. In contrast to the robust cerebro-cerebellar interactions, monosynaptically driven fMRI activation were not reliably detected. Further investigations are needed to address whether and how the functional connectivity among cortical and subcortical areas changes depending on the anesthesia state, stimulation parameters, originating cortical layer, and other unknown factors. Direct comparison of the activations induced by opto-ICMS and electrical ICMS, the latter of which is more established and generally effective to induce remote fMRI responses (Ohayon et al. 2013), would be noteworthy. Layer-specific optogenetic stimulation technique (Chan et al. 2020) or pathway-selective optogenetic/chemogenetic manipulation (Vancraeyenest et al. 2020; Inoue et al. 2021; Klink et al. 2021) would also be promising to resolve the contribution of the specific layer and type of projecting neurons to the remote activation.

## Supplementary Material

SupplementaryMaterials_tgac022

## Data Availability

Data will be made available on BALSA repositories (https://balsa.wustl.edu/study/4m5VG).
