# Supplementary material for: Cerebro-cerebellar interactions in nonhuman primates examined by optogenetic functional magnetic resonance imaging
Source: Cereb Cortex Commun. 2022 May 25;3(2):tgac022. doi: 10.1093/texcom/tgac022 (PMC9233902; doi:10.1093/texcom/tgac022)
Supplement: SupplementaryMaterials_tgac022 [file supplementarymaterials_tgac022.pdf]

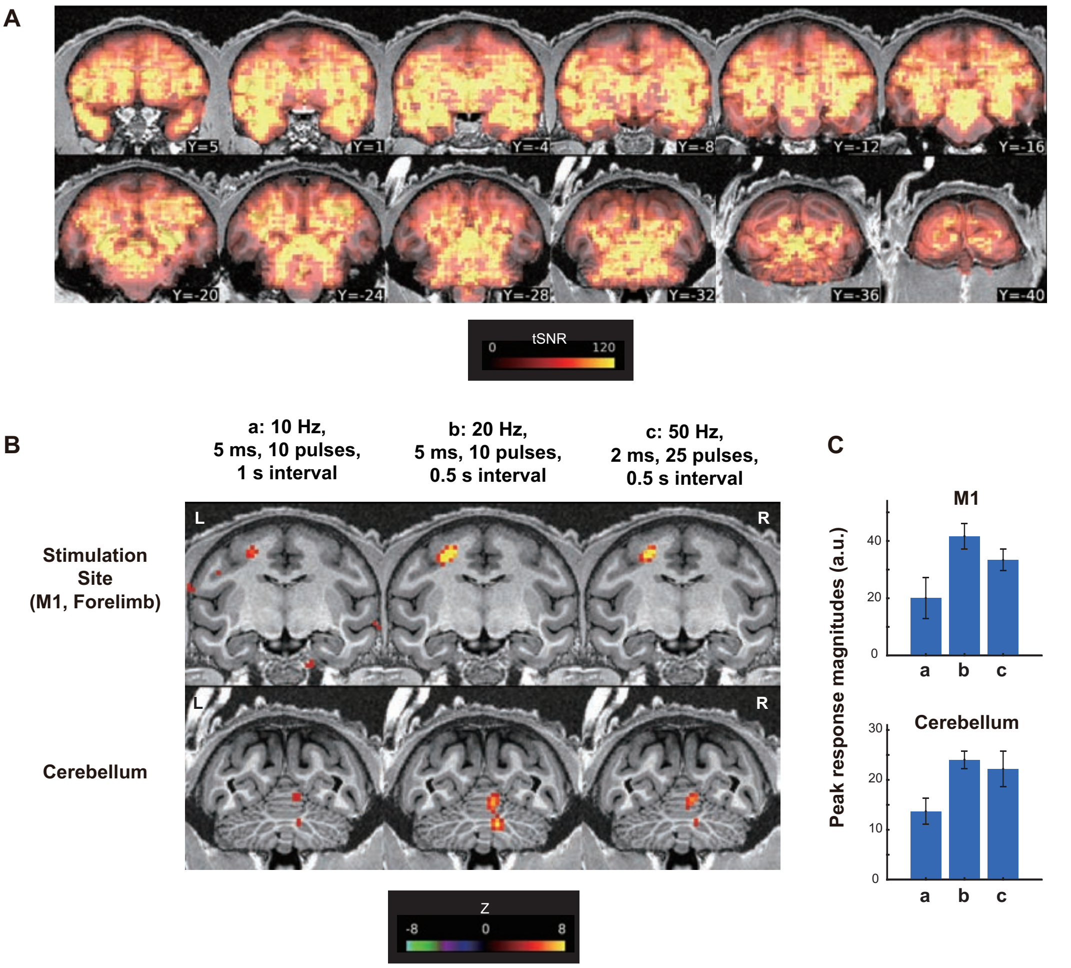

Supplementary Figure 1. Effect of the light pulse parameters on the functional magnetic resonance imaging (fMRI) activity. Results of one preliminary fMRI session for monkey C, in which the forelimb region of the primary motor cortex (M1) was optogenetically stimulated with different light pulse conditions (four runs for each condition), condition a (10 pulses of 5-ms duration at 10 Hz, interleaved with 1-s interval), condition b (10 pulses of 5-ms duration at 20 Hz with 0.5-s interval), and condition c (25 pulses of 2-ms duration at 50 Hz with 0.5-s interval). Stimulation was applied at both the distal and proximal forelimb regions simultaneously through two optical fibers (each with diameters of 100 μm). The light intensity was 15 mW. (A) A map of the temporal signal-to-noise ratio (tSNR; standard deviation divided by mean) computed from a representative fMRI run in the preliminary session (812 volumes) overlaid on the structural image. (B) *Z*-statistic color maps (stimulation versus no-stimulation, p <.001 uncorrected, two-tailed one-sample t-test) obtained in the stimulation site (upper) and cerebellar cortex (lower) with the three conditions. (C) Response magnitudes (beta weights) at the local maxima of z-statistics in the stimulation site (upper) and anterior cerebellar cortex (lower). The local maxima were estimated from the results on data from the first run for each condition (three runs in total), and the response magnitudes were evaluated using data from the rest runs (three runs for each). Error bar represents the standard error of the mean (N = 3).

The map in (A) illustrated that tSNR in the superior region around the stimulation sites and inferior-lateral region (including lateral hemispheres of the cerebellum) were low probably owing to the susceptibility-induced signal dropout and geometric distortions; however, those in the remaining parts were remarkably high (Autio et al. 2020). Regardless of the regional low tSNR and limited number of measurements, optogenetic intracortical microstimulation (opto-ICMS) with all these conditions, especially b and c, produced activations well localized in the stimulation site and the cerebellum. Note that there was no negative signal change at the stimulation site, which has been reported as a sign of thermal artifacts (Christie et al. 2013; Schmid et al. 2017; Albers et al. 2019). Thus, we considered that the thermal artifacts would be tolerable under these conditions (see also Supplementary Fig. 5C). We chose condition b as the light pulse parameter for the main fMRI experiments based on these results.

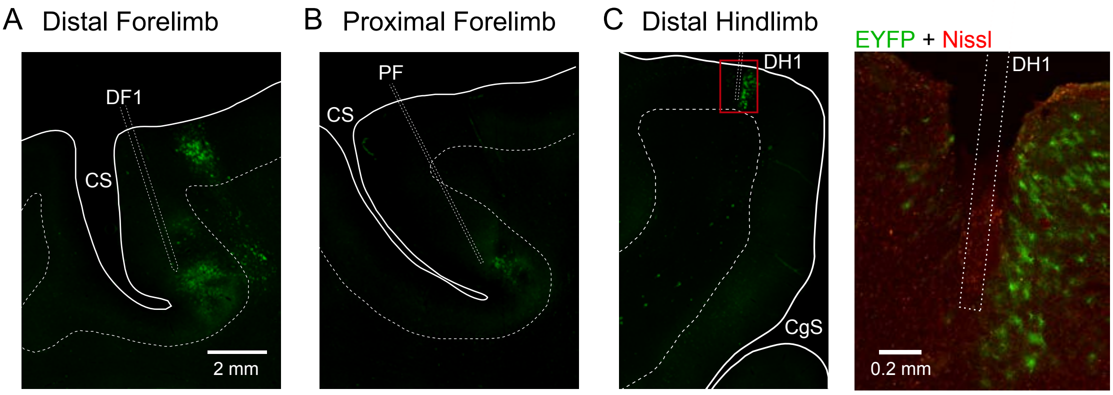

Supplementary Figure 2. Histological confirmation of adeno-associated virus (AAV) injection sites in monkey N. (A-C) Expression of hChR2(H134R)/EYFP around AAV injection sites in the distal forelimb (A), proximal forelimb (B), and distal hindlimb (C) regions of the M1 is shown in frontal sections. Dashed line rectangles indicate the presumed locations of implanted optical fibers in the distal forelimb (DF1), proximal forelimb (PF), and distal hindlimb (DH1) regions as sections that are not parallel to the optical fiber tracks (See Supplementary Table 1 for information on the implanted optical fibers). Dashed lines represent the border between gray and white matter. (A, B) Cell bodies with fluorescence signal (green) were mainly located in the deep layers, in and around the layer 5, of the M1. (C) Labeled cell bodies (green) were located in the both superficial and deep layers. The area of the red rectangle in the left is magnified in the right with Nissl stain (red). Labeled cells may contain glia, but are mainly composed of neurons based on our previous study, in which the same vector was used (Watanabe et al. 2020). CS, central sulcus; CgS, cingulate sulcus.

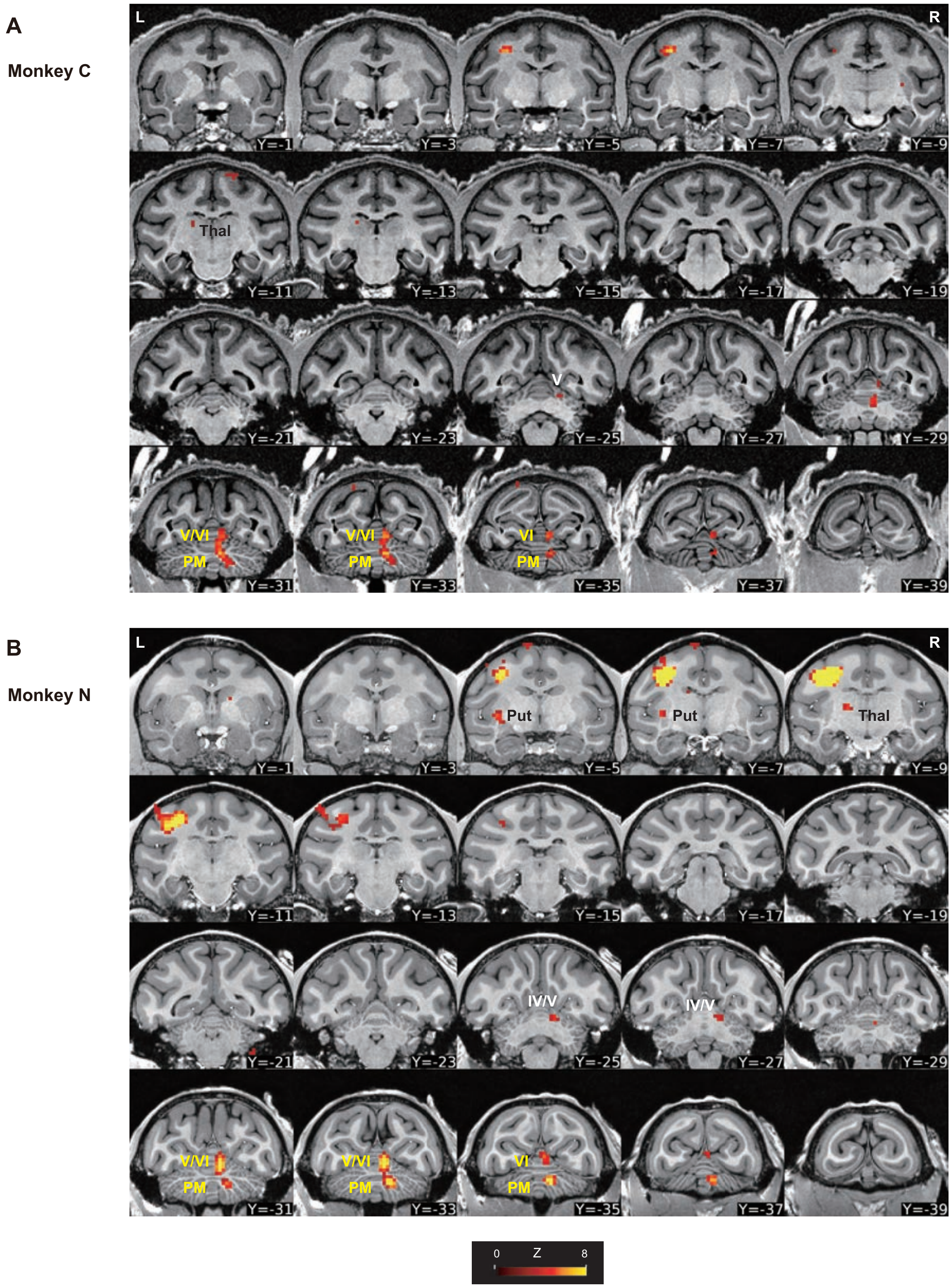

Supplementary Figure 3. Direct and remote fMRI activations evoked by opto-ICMS of the distal forelimb region of the M1. (A and B) *Z*-statistic color maps (stimulation versus no-stimulation, $p < .05$ family-wise error (FWE) corrected for multiple comparisons) were superimposed onto the coronal T1-weighted (T1w) images for monkey C (A) and monkey N (B). Letters colored with yellow and white denote the regions in the vermis and intermediate zones of the cerebellar hemispheres, respectively. IV–VI, cerebellar lobule IV–VI; Put, putamen; Thal, thalamus.

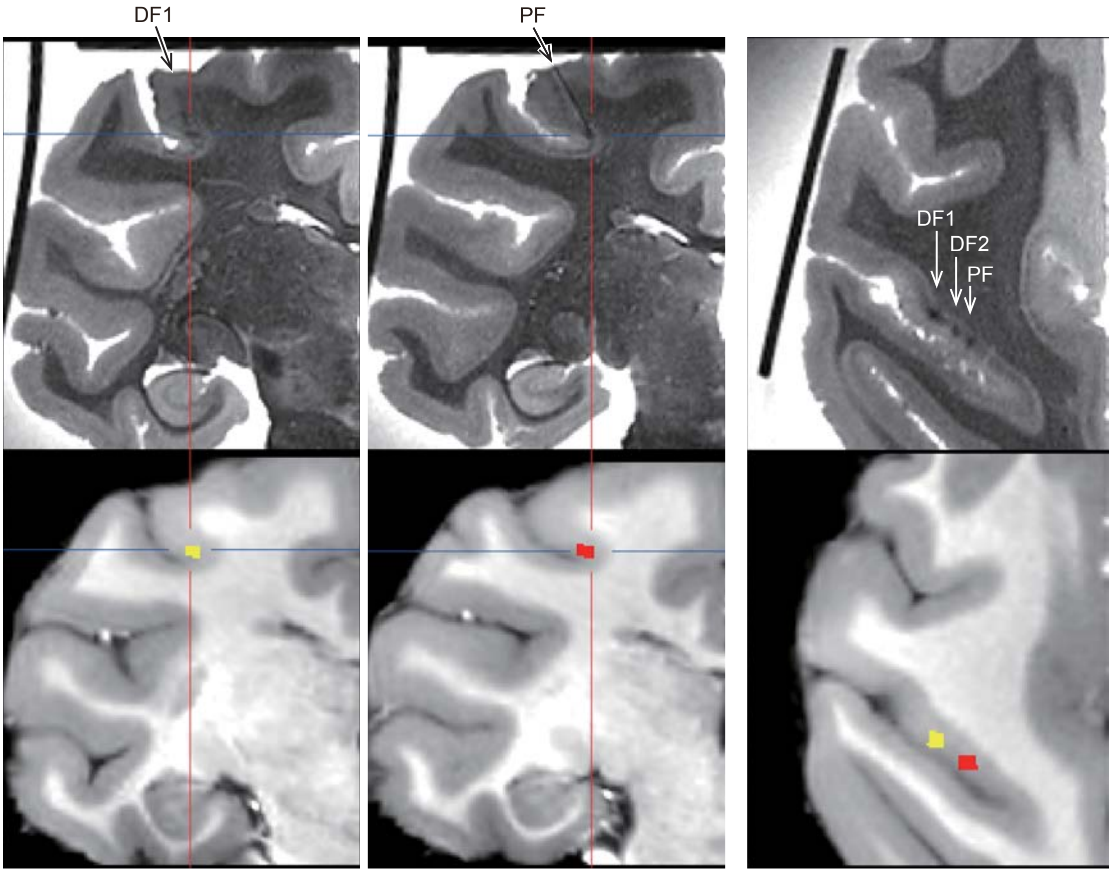

Supplementary Figure 4. Optical fiber tracks and activation peaks examined using high-resolution *ex vivo* and *in vivo* MRI in monkey N. *Ex vivo* T2-weighted (upper) and *in vivo* T1w (lower) quasicoronal images aligned parallel to the optical fiber tracks targeting the distal forelimb (DF1, left) and proximal forelimb (PF, middle) regions and axial images (right) denoted by blue horizontal lines in the left and middle panels (See Supplementary Table 1 for information on the implanted optical fibers). Yellow and red voxels (lower panels) indicate the peaks of the fMRI activation evoked by the light stimulations of the distal forelimb and proximal forelimb regions, respectively, and correspond well to the bottom ends of DF1 and PF tracks (upper panel). Another distal forelimb track in the right upper panel (DF2) was not used for the fMRI experiments.

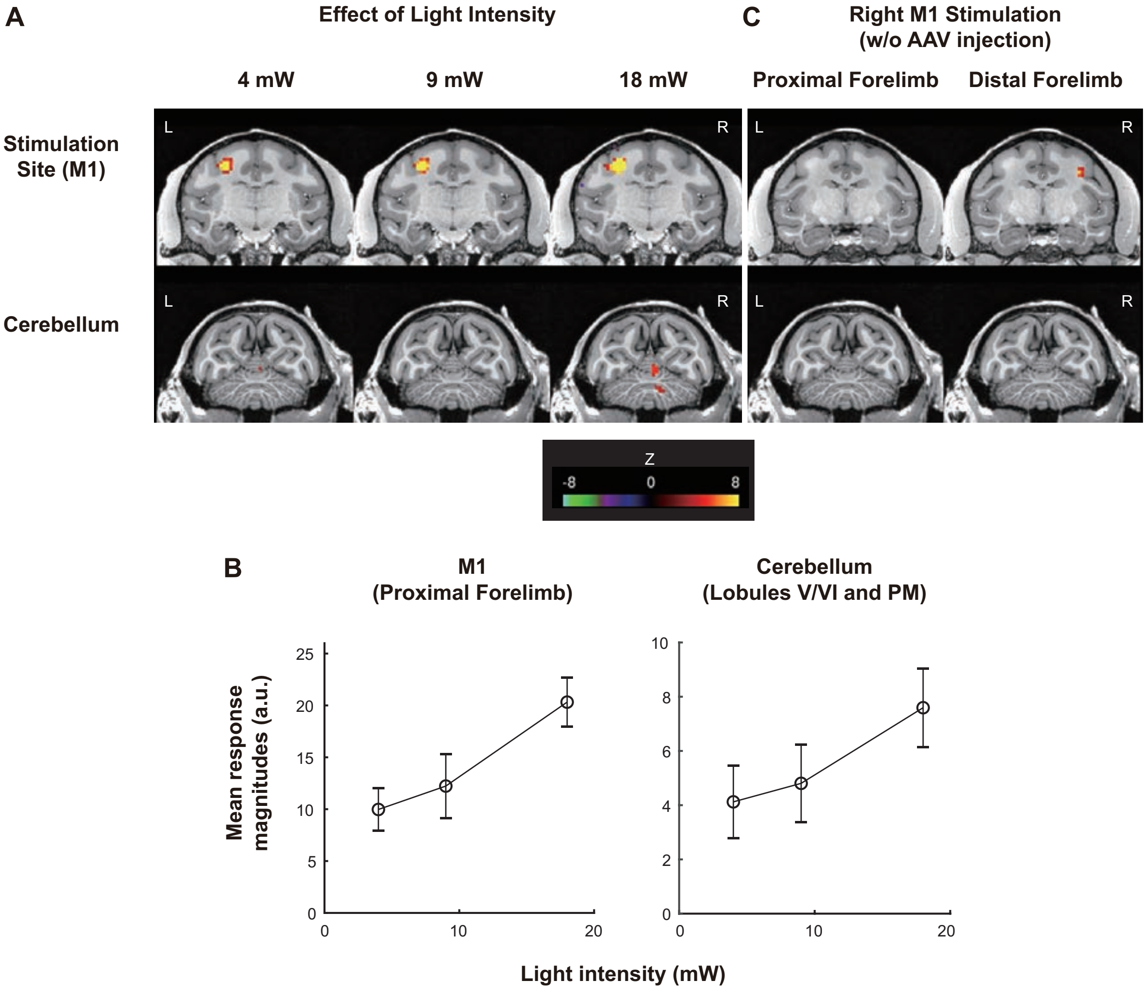

Supplementary Figure 5. Results of the control fMRI experiments. (A and B) Results of the first control fMRI experiment for monkey N that examined the effects of light intensities on the direct and remote activations. Stimulation was applied at the proximal forelimb region in the left M1 through 100FT with three light intensities (4, 9, and 18 mW; 10 pulses of 5-ms duration at 20 Hz). (A) *Z*-statistic color maps (stimulation vs no-stimulation) obtained for each intensity (p <.05, FWE-corrected for multiple comparisons, two-tailed one-sample *t*-test). (B) Mean response magnitudes (beta weights) in the M1 and cerebellar region of interest defined based on the results of the separate main fMRI experiments. Error bar represents the standard error of the mean (N=12). (C) Results of the second control fMRI experiment for monkey N that tested the effect of thermal artifacts. Stimulation (18 mW; 10 pulses of 5-ms duration at 20 Hz) was applied at the distal or proximal forelimb region of the M1 of the right hemisphere, where no viral vector was injected (12 runs for each). The M1 distal and proximal forelimb regions were stimulated through 100FT and 200CS, respectively. *Z*-statistic color maps (stimulation vs no-stimulation) were shown for each stimulation region (left, proximal forelimb; right, distal forelimb; p <.05, FWE-corrected for multiple comparisons, two-tailed one-sample *t*-test). No or only a small cluster of positive signal changes were observed at the stimulation site (C, upper panels; compare with A, upper panel, 18mW). Therefore, heat-induced signal changes at the stimulation sites (Christie et al. 2013; Schmid et al. 2017; Albers et al. 2019) would be, if any, much smaller than the optogenetic activations. Importantly, there was no evident positive or negative signal change in the remote regions (C, lower panels), confirming the absence of thermal artifacts in the cerebellum.

Supplementary Table 1. Summary of the implantation of the optical fibers.

Monkey	Hemisphere	No. of tracks	Region	Optical fiber
Monkey C	Left M1	4	Distal forelimb (wrist)	100FT
			Proximal forelimb (elbow)	100FT
			Proximal hindlimb (trunk/hip)	200FT
			Proximal hindlimb (trunk/hip)	200FT
Monkey N	Left M1	5	Distal forelimb (digit/wrist): DF1	200CS
			Distal forelimb (digit/wrist): DF2	200CS
			Proximal forelimb (elbow/shoulder): PF	100FT
			Distal hindlimb (hip/toe): DH1	100FT
			Distal hindlimb (hip/toe): DH2	200FT
	Right M1	2	Distal forelimb (digit/wrist)	100FT
	(w/o AAV injection)		Proximal forelimb (elbow/ shoulder)	200CS

Optical fibers (100FT, 100-μm core diameter with flat end; 200FT, 200-μm core diameter with flat end; 200CS, 200-μm core diameter with cone-shaped end) were implanted in the distal forelimb, proximal forelimb, proximal hindlimb, and distal hindlimb regions of the M1 after or without AAV injections based on electrophysiological mapping. DF1, DF2, PF, DH1, and DH2, optical fibers in the distal forelimb, proximal forelimb, and distal hindlimb regions shown in Supplementary Figures 2 and 4.

Supplementary References

Albers F, Wachsmuth L, Schache D, Lambers H, Faber C. 2019. Functional MRI readouts from BOLD and diffusion measurements differentially respond to optogenetic activation and tissue heating. Front Neurosci. 13:1–16.

Autio JA, Glasser MF, Ose T, Donahue CJ, Bastiani M, Ohno M, Kawabata Y, Urushibata Y, Murata K, Nishigori K, Yamaguchi M, Hori Y, Yoshida A, Go Y, Coalson TS, Jbabdi S, Sotiropoulos SN, Kennedy H, Smith S, Van Essen DC, Hayashi T. 2020. Towards HCP-Style macaque connectomes: 24-Channel 3T multi-array coil, MRI sequences and preprocessing. Neuroimage. 215:116800.

Christie IN, Wells JA, Southern P, Marina N, Kasparov S, Gourine A V., Lythgoe MF. 2013. FMRI response to blue light delivery in the naïve brain: Implications for combined optogenetic fMRI studies. Neuroimage. 66:634–641.

Schmid F, Wachsmuth L, Albers F, Schwalm M, Stroh A, Faber C. 2017. True and apparent optogenetic BOLD fMRI signals. Magn Reson Med. 77:126–136.

Watanabe H, Sano H, Chiken S, Kobayashi K, Fukata Y, Fukata M, Mushiake H, Nambu A. 2020. Forelimb movements evoked by optogenetic stimulation of the macaque motor cortex. Nat Commun. 11:1–9.
